# Cell-specific cis-natural antisense transcripts (cis-NATs) in the sperm and the pollen vegetative cells of *Arabidopsis thaliana*

**DOI:** 10.12688/f1000research.13311.1

**Published:** 2018-01-22

**Authors:** Peng Qin, Ann E. Loraine, Sheila McCormick

**Affiliations:** 1 Rice Research Institute, Sichuan Agricultural University, Chengdu Wenjiang, Sichuan, 611130, China; 2 U.S. Department of Agriculture/Agricultural Research Service and Department of Plant and Microbial Biology, University of California, Berkeley, Albany, CA, 94710, USA; 3 Department of Bioinformatics and Genomics, University of North Carolina at Charlotte, Charlotte, NC, 28223, USA

**Keywords:** Pollen, Sperm, Cis-Natural antisense transcript, Arabidopsis

## Abstract

**Background: **cis-NATs **(**cis-natural antisense transcripts **)** are transcribed from opposite strands of adjacent genes and have been shown to regulate gene expression by generating small RNAs from the overlapping region. cis-NATs are important for plant development and resistance to pathogens and stress. Several genome-wide investigations identified a number of cis-NAT pairs, but these investigations predicted cis-NATS using expression data from bulk samples that included lots of cell types. Some cis-NAT pairs identified from those investigations might not be functional, because both transcripts of cis-NAT pairs need to be co-expressed in the same cell. Pollen only contains two cell types, two sperm and one vegetative cell, which makes cell-specific investigation of cis-NATs possible.

**Methods: **We investigated potential protein-coding cis-NATs in pollen and sperm using pollen RNA-seq data and TAIR10 gene models using the Integrated Genome Browser.  We then used sperm microarray data and sRNAs in sperm and pollen to determine possibly functional cis-NATs in the sperm or vegetative cell, respectively.

**Results: **We identified 1471 potential protein-coding cis-NAT pairs, including 131 novel pairs that were not present in TAIR10 gene models. In pollen, 872 possibly functional pairs were identified. 72 and 56 pairs were potentially functional in sperm and vegetative cells, respectively. sRNAs were detected at 794 genes, belonging to 739 pairs.

**Conclusion: **These potential candidates in sperm and the vegetative cell are tools for understanding gene expression mechanisms in pollen.

## Introduction

Natural antisense transcripts (NATs) are endogenous transcripts that contain sequences complementary to each other. NATs have been shown to regulate gene expression by generating small RNAs from the overlapping region ( Zhang *et al.*, 2013). NATs are classified into two subgroups according to the site of their biogenesis: trans-NATs and cis-NATs. Trans-NATs are transcribed from different genomic loci, whereas cis-NATs are transcribed from opposite strands of adjacent genes ( Jin *et al.*, 2008). Based on the relative orientation and overlap degree of two transcripts, cis-NATs can be categorized into three types: head-to-head (5′ to 5′), tail-to-tail (3′ to 3′) and fully overlapping ( Jin *et al.*, 2008). cis-NATs are widely present in plants, animals and fungi ( Zhang *et al.*, 2013). In plants, cis-NATs are important for pathogen resistance ( Katiyar-Agarwal *et al.*, 2006), stress tolerance ( Borsani *et al.*, 2005), successful fertilization ( Ron *et al.*, 2010), and phosphate homeostasis and plant fitness ( Jabnoune *et al.*, 2013).

Several genome-wide investigations identified potential cis-NATs in plants, ranging from 1057 to 1710 pairs in *Arabidopsis* ( Henz *et al.*, 2007; Jin *et al.*, 2008; Wang *et al.*, 2005; Zhan & Lukens 2013), and 3819 pairs in rice ( Lu *et al.*, 2012). However, all the expression data used in these cis-NAT investigations were from bulk samples such as seedlings, leaves or inflorescences, which include many cell types. For potential cis-NATs to be functionally relevant, the reverse and complementary transcripts must be co-expressed in the same cell. Some potential cis-NATs identified in those investigations might be expressed in different cells and, thus, the presence of overlapping transcripts in the same cell is not known. Moreover, the regions predicted to overlap in previous investigations were based on available gene model annotations, which might not fully represent potential overlaps, due to alternative splicing at different developmental stages or, to more extensive 5’ or 3’ UTRs than are annotated.

A pollen grain contains only two types of cell, one vegetative cell and two sperm cells, and thus can be used to investigate cell-specific cis-NAT pairs. Pollen RNA-seq data ( Loraine *et al.*, 2013) provides accurate transcript lengths, helpful for precisely identifying overlapping regions of two adjacent genes. Sperm microarray data ( Borges *et al.*, 2008) is helpful for defining whether two adjacent genes are expressed in the same cell. Moreover, a small RNA database for pollen and sperm ( Slotkin *et al.*, 2009) can be used to determine if small RNAs were detected from any overlapping regions.

In this study, we investigated potential cis-NATs in *Arabidopsis* sperm and vegetative cells using pollen RNA-seq data, sperm microarray data and sRNA data in pollen and sperm. In total, we identified 1471 cis-NAT pairs, including 131 novel pairs, with 72 and 56 pairs being potentially functional cis-NATs in sperm and vegetative cells respectively. These cis-NATs are tools for understanding gene regulation mechanisms in sperm and vegetative cells.

## Methods

We investigated potential cis-NATs from protein-coding genes in pollen and sperm using pollen RNA-seq data ( Borges *et al.*, 2008; Loraine *et al.*, 2013) and TAIR10 gene models (TAIR10 gene annotation data available here) using the Integrated Genome Browser, available from http://www.bioviz.org ( Freese *et al.*, 2016). We loaded TAIR10 gene models s and pollen RNA-seq into IGB, then manually scanned for cis-NATs in each chromosome, based on the following parameters: 1) the orientation of two adjacent genes in TAIR10 was reverse and complementary; 2) the length of transcripts mapping to the overlap of two adjacent genes was larger than 21nt, because the size of sRNA generated by cis-NATs is normally larger than 21 nt; and 3) both adjacent genes encoded proteins. The expression and sRNA data of each cis-NAT was merged with the cis-NAT data in Excel to generate sheet 1 of Table S1. Different categories of cis-NATs in sheets 2–8 of Table S1 were obtained based on the cis-NAT data in sheet 1 of Table S1.

## Results

**Figure 1.**
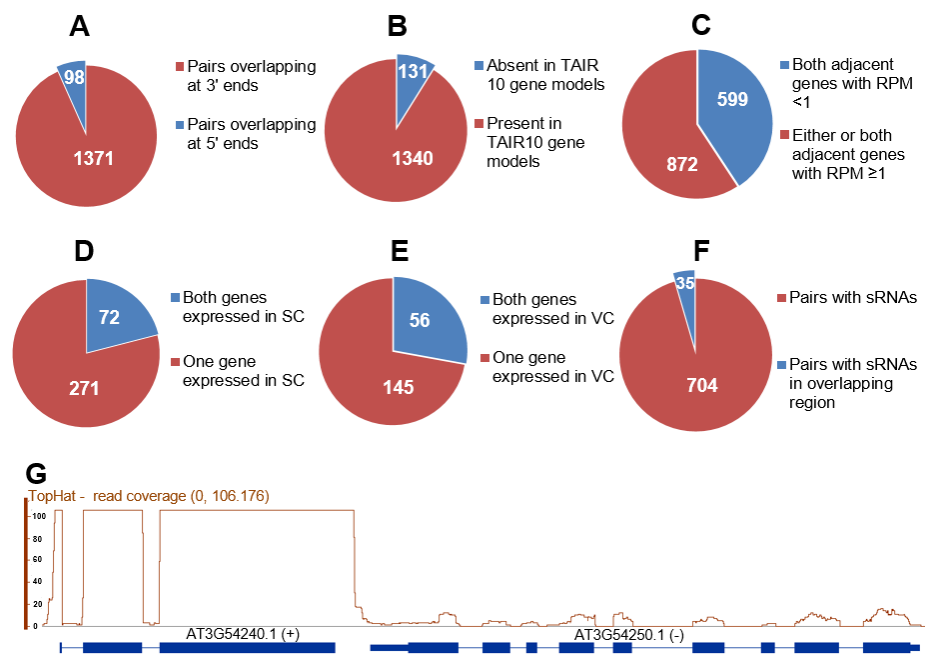
Overview of potential cis-NATs in sperm and the pollen vegetative cell. ( **A**) Overview of potential cis-NATs with different overlapping types. ( **B**) Overview of potential cis-NATs present or non-present in TAIR 10 gene models. ( **C**) Overview of potential cis-NATs based on reads per million (RPM) from pollen RNA-seq data. ( **D**) Overview of potential cis-NATs possibly functional in sperm cells (SC). ( **E**) Overview of potential cis-NATs possibly functional in the vegetative cell (VC). ( **F**) Overview of potential cis-NATs with sRNAs in vegetative and sperm cells.( **G**) Potential cis-NATs not present in TAIR10 gene models, but detected in pollen RNA-seq data.

We identified 1471 potential cis-NAT pairs, comprising 1373 pairs whose transcripts were complementary at their 3’ ends, and 98 pairs whose transcripts were complementary at their 5’ ends ( Figure 1A, sheet 1 and 2 of Table S1). Among these 1471, in 37 pairs one transcript was completely internal to the other, 100 pairs comprised 50 sets that had three overlapping genes (sheet 1 of Table S1), and 131 pairs (8.9%) were not apparent using the TAIR 10 gene models, but were detected in the pollen RNA-seq data ( Figure 1B and G, sheet 1 of Table S1).

One criterion for functional cis-NATs is that the two adjacent genes of a cis-NAT pair are expressed. To identify potentially functional cis-NATs in pollen, we analyzed the expression level of the 1471 gene pairs, defining genes with reads per million (RPM) ≥1 as expressed. There were 599 pairs for which the RPM of two adjacent genes was lower than 1 (sheet 3 of Table S1), suggesting that those 599 pairs might not produce relevant cis-NATs in pollen. Most sperm-specific genes, such as *GEX1*, *GEX2* ( Rutley & Twell, 2015), and *Kokopelli* ( Ron *et al.*, 2010), are detectable in pollen RNA-seq data, but sperm-specific genes with lower expression levels, such as *ARI14*, might not be detected, as the proportion of RNA from the vegetative cell is much larger than that from the sperm cells. So potential cis-NATs with one expressed gene in pollen RNA-seq data might still be functional in pollen. Based on this, there were 872 possibly functional cis-NATs pairs, for which the RPM of either or both adjacent genes was ≥1 ( Figure 1C, sheet 4 of Table S1), of which 62 pairs did not overlap in TAIR10 gene models. Note that we did not detect a cis-NAT pair between *Kokopelli* and *ARI14* ( Ron *et al.*, 2010), because the TAIR 10 gene model does not show them overlapping, and *ARI14* expression is low in wild type. Thus it is possible that other cis-NATs might similarly not be included in sheet 4 of Table S1 (see below).

Another criterion for functional cis-NATs is that both adjacent genes are expressed in the same cell. In order to accurately identify potentially functional cis-NATs in pollen, we investigated the gene expression level of 872 pairs in sperm cells. The microarray signal calls “Present (P) or Absent (A)” ( Borges *et al.*, 2008) were used to categorize genes as expressed or not expressed, respectively. We identified 72 pairs for which both adjacent genes were expressed in sperm, supporting the likelihood that these 72 cis-NATs pairs exist in sperm (sheet 5 of Table S1). There were an additional 271 pairs for which only one of the adjacent genes was expressed in sperm ( Figure 1D and sheet 6 of Table S1), some of which might pertain to the *Kokopelli*/ *ARI14* example. To test if these pairs might function in the vegetative cell, we defined the gene as expressed in the vegetative cell if either 1): both pollen and sperm signals were called “P”, and the ratio of the pollen to sperm signal was > 3; or 2): the pollen signal was “P” and sperm signal was “A”. This exercise yielded 56 pairs for which both adjacent genes were expressed in the vegetative cell (sheet 7 of Table S1), and 145 pairs for which only one was expressed in the vegetative cell ( Figure 1E and sheet 8 of Table S1). Another hallmark of functional cis-NATs is that there are small RNAs generated from the overlapping region. We therefore investigated the sRNAs of pollen and sperm ( Slotkin *et al.*, 2009) for these potentially functional cis-NAT pairs. sRNAs were detected at 794 genes, belonging to 739 pairs ( Figure 1F and sheet 1 of Table S1). Of these, 35 cis-NATs pairs had sRNAs from the overlapping region.

## Discussion

cis-NATs are widely present in plants, and play an important role in regulating gene expression. However, in plants precise identification of cis-NATs at a cell-specific level to support whether cis-NATs might be functional is lacking. One possible reason was that it is difficult to get specific cell types for RNA-seq. As pollen grains contain only two types of cell, they are an excellent model to investigate cell-specific cis-NATs.

The vegetative cell forms a pollen tube that transports two sperm cells into the ovule for fertilization. Successful fertilization needs proper gene regulation in both the vegetative and sperm cells ( Ron *et al.*, 2010). The precise identification of cis-NATs in pollen is a tool for understanding the molecular mechanism of pollen tube growth and fertilization. The 131 novel potential cis-NATs in pollen, and the 72 and 56 potentially functional cis-NATs in sperm and the vegetative cell, respectively, provide candidates toward further uncovering the regulatory mechanisms of gene expression in the sperm and vegetative cells.

## Conclusion

We identified 1471 potential cis-NAT pairs, including 131 pairs only detected in the pollen RNA-seq data. There were 872 pairs expressed in the same cell and thus possibly functional in pollen, while 72 and 56 pairs were potentially functional in sperm and vegetative cell, respectively.

## Data availability

*Arabidopsis* pollen RNA-seq alignments data were loaded into Integrated Genome Browser from the IGB Quickload site

Unprocessed sequence data are available from the Sequence Read Archive under accession SRP022162 ( Loraine *et al.*, 2013).

The pollen and sperm microarray data was from ( Borges *et al.*, 2008) (available at https://bmcplantbiol.biomedcentral.com/articles/10.1186/1471-2229-9-87)

sRNAs in *Arabidopsis* pollen and sperm were downloaded from https://mpss.danforthcenter.org/dbs/index.php?SITE=at_sRNA&lib_type=sRNA&lib_id=487 and https://mpss.danforthcenter.org/dbs/index.php?SITE=at_sRNA&lib_type=sRNA&lib_id=489, respectively ( Slotkin *et al.*, 2009).

## Supplemental material

**Table S1.** Investigation of cis-NATs in pollen and sperm cells of *Arabidopsis*.

Sheet 1) List of all potential cis-NATs identified using TAIR 10 gene models and pollen RNA-seq data; Sheet 2) List of potential cis-NAT pairs with three overlapping genes; Sheet 3) List of potential cis-NATs pairs for which the RPM of both adjacent genes is < 1; Sheet 4) List of potential cis-NATs pairs for which the RPM of either or both adjacent genes is ≥ 1; Sheet 5) List of potential cis-NAT pairs for which both adjacent genes are expressed in sperm cells; Sheet 6) List of potential cis-NAT pairs for which only one gene is expressed in sperm cells; Sheet 7) List of potential cis-NAT pairs for which both adjacent genes are expressed in the vegetative cell; Sheet 8) List of potential cis-NAT pairs for which only one gene is expressed in the vegetative cell.
