# Supplementary material for: Cell-specific cis-natural antisense transcripts (cis-NATs) in the sperm and the pollen vegetative cells of Arabidopsis thaliana
Source: F1000Res. 2018 Jan 22;7:93. [Version 1] doi: 10.12688/f1000research.13311.1 (PMC5946162; doi:10.12688/f1000research.13311.1)
Supplement: Peer review history

# Peer review: Referee response for version 1

**DOI:** 10.5256/f1000research.14444.r33655

**Published:** 2018-05-16

**Authors:** Jules Deforges

**Affiliations:** 1 CNRS (French National Center for Scientific Research), UMR 8015, Laboratory of Crystallography and NMR Biology, Paris Descartes University, Paris, France

In this study, the authors have addressed an important question often neglected regarding the regulation of gene expression by cis-Natural Antisense Transcripts. Cis-NATs usually identified by sequencing of tissues containing many different cell types are assumed to potentially regulate their cognate coding genes co-expressed in the same tissue. However, direct evidence that both transcripts of the pairs are actually expressed in the same cells is often missing.

 By taking advantage of RNAseq, micro-array as well as sRNA data from pollen, a tissue containing only 2 cell types, the authors identified 72 cis-NAT pairs for which both transcripts were expressed in Sperm cells and 56 in vegetative cells. In addition, they identified sRNA falling in the overlapping region of 35 cis-NAT pairs out of 739 in pollen, providing insight about the functional role of these cis-NATs.

 Qin et al. provide useful data about cis-NAT expression in sperm and vegetative cells in pollen. They also provide insight about their potential function in sRNA production, contributing to a better understanding of the mechanisms of gene regulation by cis-NATs.

I have read this submission. I believe that I have an appropriate level of expertise to confirm that it is of an acceptable scientific standard.

# Peer review: Referee response for version 1

**DOI:** 10.5256/f1000research.14444.r33361

**Published:** 2018-05-10

**Authors:** Robert A. Martienssen, Cristiane Alves

**Affiliations:** 1 Department of Plant Genetics, Cold Spring Harbor Laboratory, Cold Spring Harbor, NY, 11724, USA; 2 Cold Spring Harbor Laboratory, Cold Spring Harbor, NY, USA

The have authors addressed an interesting question regarding gene regulation in vegetative and sperm cells in pollen by cis-natural antisense transcripts (cis-NATs). Qin et al. explain how cis-NATs are generated and their importance for plant development, pathogen resistance, stress response, homeostasis and fertilization. The main question for this work regards the discrepancy that may be occurring in previously published data, as most analysis were performed on bulked samples containing different types of cells leading to possible misassignment of cis-NAT pairing. cis-NATs should be co-expressed in the same cell, as the authors described, so the analysis should be done at the least on the same cell type. To address this issue, the authors used RNA-seq and microarray publicly available data from vegetative and sperm cells from *Arabidopsis thaliana* pollen in order to investigate cell-specific cis-NATs pairs, using specific parameters avoiding possible mispairing. The authors identified possibly functional cis-NATs in pollen, 56 pairs in the vegetative cell and 72 in sperm cells, also, the authors screened for small RNAs generated from the pairing regions, finding that 35 out of 739 pollen cis-NATs from the overlapping region, suggesting a regulatory function. It would be interesting to summarize in the text if any of these were in sperm vs VN.

 Function analysis is yet to be addressed, as the authors pointed out, to confirm the biological role of cis-NATs in plant development, stress response, pathogen resistance, homeostasis and fertilization. But the authors choice to survey pollen grains where cell specificity is known could be key to understanding molecular mechanisms regarding fertilization and heredity.

We have read this submission. We believe that we have an appropriate level of expertise to confirm that it is of an acceptable scientific standard.

# Peer review: Referee response for version 1

**DOI:** 10.5256/f1000research.14444.r30668

**Published:** 2018-03-21

**Authors:** Hailing Jin

**Affiliations:** 1 Department of Plant Pathology and Microbiology, Institute for Integrative Genome Biology, University of California, Riverside, Riverside, CA, USA

This manuscript by Qin et al. addresses an important question in the gene regulation field: How cis-natural antisense transcripts (cis-NATs) are co-regulated within the same cell. Although many genome-wide studies on cis-NATs have been reported in different species, most expression analysis was based on data from bulk samples that include many different cell types. The conclusions from those studies may not be entirely accurate because it is not clear if the two transcripts of each cis-NAT pair indeed express within the same cell. The authors of this study used a unique system – pollen for the analysis because pollen only has two cell types, sperm and vegetative cells, which allows easy identification of NATs that expressed in the same cell type. They identified 872 potential functional cis-NAT pairs in pollen. Microarray expression analysis identified 72 and 56 pairs were potentially functional in sperm and vegetative cells, respectively, as both adjacent genes were expressed. In addition, 271 pairs and 145 pairs for which only one was expressed in sperm or vegetative cell, respectively. It is possible that one of the transcripts within a pair that is expressed may suppress the expression of the other transcript in the same cell. Furthermore, 739 pairs of cis-NATs generated sRNAs and 35 cis-NATs pairs had sRNAs from the overlapping region, suggesting that sRNAs play a role in the co-regulation of NAT genes.

 Co-regulation analysis of cis-NATs within the same cell is an important but often overlooked criteria, this study provided an excellent example to take the localization of gene expression into consideration, which not only identified a set of pollen-specific functional cis-NATs and sRNAs for future study, but also help us understand the regulatory mechanism of cis-NATs. Thus, I support this manuscript to be indexed without delay.

I have read this submission. I believe that I have an appropriate level of expertise to confirm that it is of an acceptable scientific standard.
